# Supplementary material for: MetaQTL: a package of new computational methods for the meta-analysis of QTL mapping experiments
Source: BMC Bioinformatics. 2007 Feb 8;8:49. doi: 10.1186/1471-2105-8-49 (PMC1808479; doi:10.1186/1471-2105-8-49)
Supplement: Additional File 7 — MetaQTL Package : jar file and tutorial. This Zip archive contains both the MetaQTL JAR file and the files of the tutorial. [file 1471-2105-8-49-S7.zip › org.inra.metaqtl/doc/org/metaqtl/graph/class-use/ChromGraph.html]

Uses of Class org.metaqtl.graph.ChromGraph


|  |  |  |
| --- | --- | --- |
| **Overview** \| **Package** \| **Class** \| **Use** \| **Tree** \| **Deprecated** \| **Index** \| **Help** | |  |
| PREV   NEXT | **FRAMES**    **NO FRAMES**     **All Classes** |  |


---


## **Uses of Class org.metaqtl.graph.ChromGraph**

| Packages that use ChromGraph | |
| --- | --- |
| **org.metaqtl.graph** |  |
| **org.metaqtl.main** |  |

Uses of ChromGraph in org.metaqtl.graph

| Methods in org.metaqtl.graph with parameters of type ChromGraph | |
| --- | --- |
| `static java.awt.image.BufferedImage` | `GraphFactory.getImage(ChromGraph[] chroms)` |
| `void` | `MetaGraph.init(java.awt.Graphics2D graph, ChromGraph[] maps)` |

Uses of ChromGraph in org.metaqtl.main

| Methods in org.metaqtl.main that return ChromGraph | |
| --- | --- |
| `static ChromGraph[]` | `MetaMain.getChromGraph(Chromosome[] chroms, boolean withQtl, int modeQtl, IBioOntology ontology, MetaQtlAnalysis treeAnalysis)` |

| Methods in org.metaqtl.main with parameters of type ChromGraph | |
| --- | --- |
| `static void` | `MetaMain.synchChromGraphs(ChromGraph[] chromGraphs, java.util.Set partNames)` |
| `static void` | `MetaMain.writeImg(ChromGraph[] chroms, java.io.File file, java.lang.String imgFormat)` |

---


|  |  |  |
| --- | --- | --- |
| **Overview** \| **Package** \| **Class** \| **Use** \| **Tree** \| **Deprecated** \| **Index** \| **Help** | |  |
| PREV   NEXT | **FRAMES**    **NO FRAMES**     **All Classes** |  |


---
